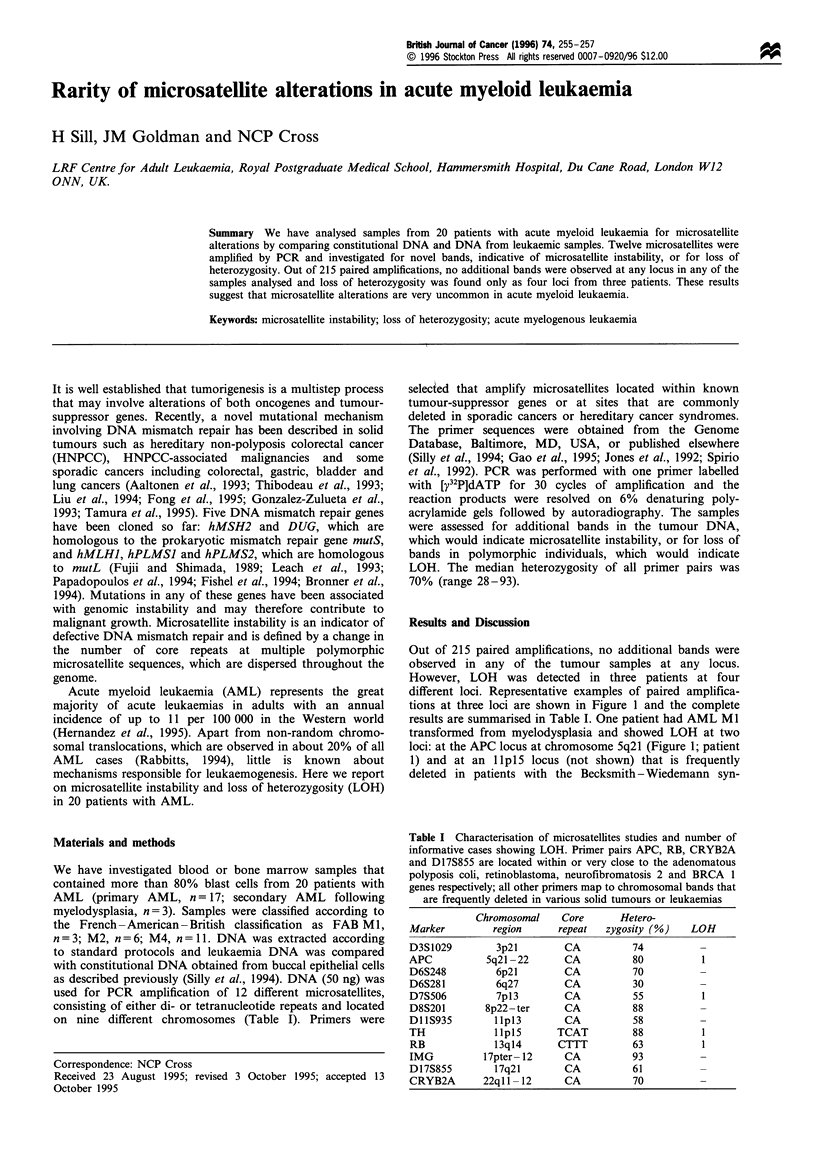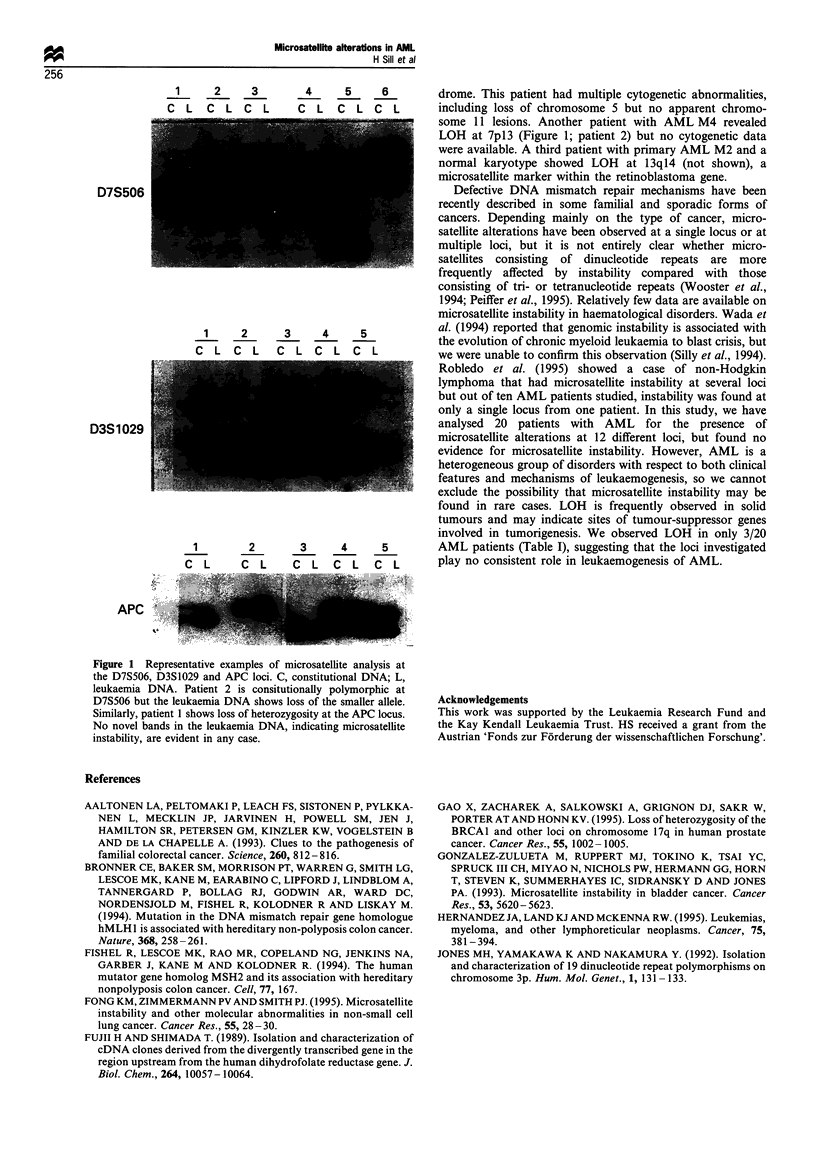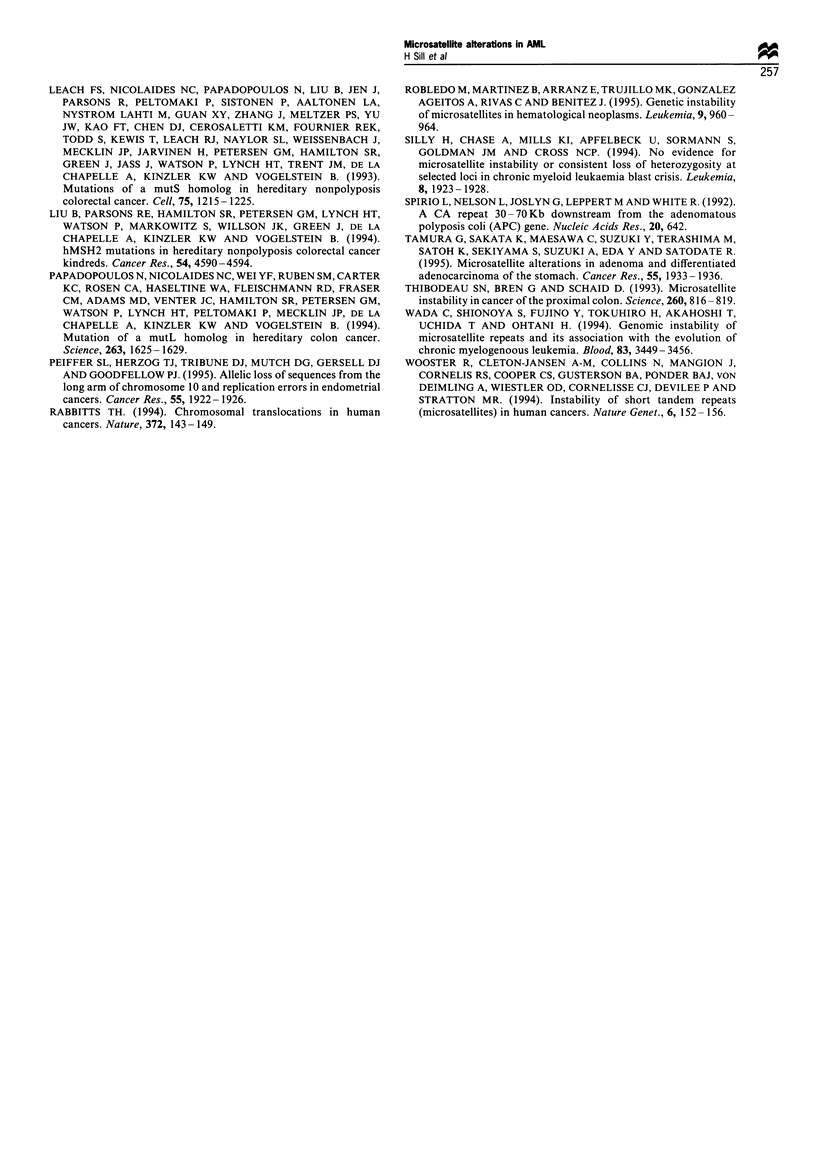# Rarity of microsatellite alterations in acute myeloid leukaemia.

**DOI:** 10.1038/bjc.1996.347

**Published:** 1996-07

**Authors:** H. Sill, J. M. Goldman, N. C. Cross

**Affiliations:** LRF Centre for Adult Leukaemia, Royal Postgraduate Medical School, Hammersmith Hospital, London, UK.

## Abstract

**Images:**


British Journal of Cancer (1996) 74, 255-257
© 1996 Stockton Press All rights reserved 0007-0920/96 $12.00

### Rarity of microsatellite alterations in acute myeloid leukaemia

H Sill, JM Goldman and NCP Cross

*LRF Centre for Adult Leukaemia, Royal Postgraduate Medical School, Hammersmith Hospital, Du Cane Road, London W12 ONN, UK.*

**Summary** We have analysed samples from 20 patients with acute myeloid leukaemia for microsatellite alterations by comparing constitutional DNA and DNA from leukaemic samples. Twelve microsatellites were amplified by PCR and investigated for novel bands, indicative of microsatellite instability, or for loss of heterozygosity. Out of 215 paired amplifications, no additional bands were observed at any locus in any of the samples analysed and loss of heterozygosity was found only as four loci from three patients. These results suggest that microsatellite alterations are very uncommon in acute myeloid leukaemia.

**Keywords:** microsatellite instability; loss of heterozygosity; acute myelogenous leukaemia

It is well established that tumorigenesis is a multistep process that may involve alterations of both oncogenes and tumour-suppressor genes. Recently, a novel mutational mechanism involving DNA mismatch repair has been described in solid tumours such as hereditary non-polyposis colorectal cancer (HNPCC), HNPCC-associated malignancies and some sporadic cancers including colorectal, gastric, bladder and lung cancers (Aaltonen *et al.*, 1993; Thibodeau *et al.*, 1993; Liu *et al.*, 1994; Fong *et al.*, 1995; Gonzalez-Zulueta *et al.*, 1993; Tamura *et al.*, 1995). Five DNA mismatch repair genes have been cloned so far: *hMSH2* and *DUG*, which are homologous to the prokaryotic mismatch repair gene *mutS*, and *hMLH1*, *hPLMS1* and *hPLMS2*, which are homologous to *mutL* (Fujii and Shimada, 1989; Leach *et al.*, 1993; Papadopoulos *et al.*, 1994; Fishel *et al.*, 1994; Bronner *et al.*, 1994). Mutations in any of these genes have been associated with genomic instability and may therefore contribute to malignant growth. Microsatellite instability is an indicator of defective DNA mismatch repair and is defined by a change in the number of core repeats at multiple polymorphic microsatellite sequences, which are dispersed throughout the genome.

Acute myeloid leukaemia (AML) represents the great majority of acute leukaemias in adults with an annual incidence of up to 11 per 100 000 in the Western world (Hernandez *et al.*, 1995). Apart from non-random chromosomal translocations, which are observed in about 20% of all AML cases (Rabbitts, 1994), little is known about mechanisms responsible for leukaemogenesis. Here we report on microsatellite instability and loss of heterozygosity (LOH) in 20 patients with AML.

### Materials and methods

We have investigated blood or bone marrow samples that contained more than 80% blast cells from 20 patients with AML (primary AML, $n=17$; secondary AML following myelodysplasia, $n=3$). Samples were classified according to the French–American–British classification as FAB M1, $n=3$; M2, $n=6$; M4, $n=11$. DNA was extracted according to standard protocols and leukaemia DNA was compared with constitutional DNA obtained from buccal epithelial cells as described previously (Silly *et al.*, 1994). DNA (50 ng) was used for PCR amplification of 12 different microsatellites, consisting of either di- or tetranucleotide repeats and located on nine different chromosomes (Table I). Primers were selected that amplify microsatellites located within known tumour-suppressor genes or at sites that are commonly deleted in sporadic cancers or hereditary cancer syndromes. The primer sequences were obtained from the Genome Database, Baltimore, MD, USA, or published elsewhere (Silly *et al.*, 1994; Gao *et al.*, 1995; Jones *et al.*, 1992; Spirio *et al.*, 1992). PCR was performed with one primer labelled with [$\gamma^{32}$P]dATP for 30 cycles of amplification and the reaction products were resolved on 6% denaturing polyacrylamide gels followed by autoradiography. The samples were assessed for additional bands in the tumour DNA, which would indicate microsatellite instability, or for loss of bands in polymorphic individuals, which would indicate LOH. The median heterozygosity of all primer pairs was 70% (range 28–93).

### Results and Discussion

Out of 215 paired amplifications, no additional bands were observed in any of the tumour samples at any locus. However, LOH was detected in three patients at four different loci. Representative examples of paired amplifications at three loci are shown in Figure 1 and the complete results are summarised in Table I. One patient had AML M1 transformed from myelodysplasia and showed LOH at two loci: at the APC locus at chromosome 5q21 (Figure 1; patient 1) and at an 11p15 locus (not shown) that is frequently deleted in patients with the Becksmith–Wiedemann syn-

**Table I** Characterisation of microsatellites studies and number of informative cases showing LOH. Primer pairs APC, RB, CRYB2A and D17S855 are located within or very close to the adenomatous polyposis coli, retinoblastoma, neurofibromatosis 2 and BRCA 1 genes respectively; all other primers map to chromosomal bands that are frequently deleted in various solid tumours or leukaemias

| *Marker* | *Chromosomal region* | *Core repeat* | *Hetero-zygosity (%)* | *LOH* |
|---|---|---|---|---|
| D3S1029 | 3p21 | CA | 74 | – |
| APC | 5q21–22 | CA | 80 | 1 |
| D6S248 | 6p21 | CA | 70 | – |
| D6S281 | 6q27 | CA | 30 | – |
| D7S506 | 7p13 | CA | 55 | 1 |
| D8S201 | 8p22–ter | CA | 88 | – |
| D11S935 | 11p13 | CA | 58 | – |
| TH | 11p15 | TCAT | 88 | 1 |
| RB | 13q14 | CTTT | 63 | 1 |
| IMG | 17pter–12 | CA | 93 | – |
| D17S855 | 17q21 | CA | 61 | – |
| CRYB2A | 22q11–12 | CA | 70 | – |

Correspondence: NCP Cross
Received 23 August 1995; revised 3 October 1995; accepted 13 October 1995

**Figure 1** Representative examples of microsatellite analysis at the D7S506, D3S1029 and APC loci. C, constitutional DNA; L, leukaemia DNA. Patient 2 is consitutionally polymorphic at D7S506 but the leukaemia DNA shows loss of the smaller allele. Similarly, patient 1 shows loss of heterozygosity at the APC locus. No novel bands in the leukaemia DNA, indicating microsatellite instability, are evident in any case.

drome. This patient had multiple cytogenetic abnormalities, including loss of chromosome 5 but no apparent chromosome 11 lesions. Another patient with AML M4 revealed LOH at 7p13 (Figure 1; patient 2) but no cytogenetic data were available. A third patient with primary AML M2 and a normal karyotype showed LOH at 13q14 (not shown), a microsatellite marker within the retinoblastoma gene.

Defective DNA mismatch repair mechanisms have been recently described in some familial and sporadic forms of cancers. Depending mainly on the type of cancer, microsatellite alterations have been observed at a single locus or at multiple loci, but it is not entirely clear whether microsatellites consisting of dinucleotide repeats are more frequently affected by instability compared with those consisting of tri- or tetranucleotide repeats (Wooster *et al.*, 1994; Peiffer *et al.*, 1995). Relatively few data are available on microsatellite instability in haematological disorders. Wada *et al.* (1994) reported that genomic instability is associated with the evolution of chronic myeloid leukaemia to blast crisis, but we were unable to confirm this observation (Silly *et al.*, 1994). Robledo *et al.* (1995) showed a case of non-Hodgkin lymphoma that had microsatellite instability at several loci but out of ten AML patients studied, instability was found at only a single locus from one patient. In this study, we have analysed 20 patients with AML for the presence of microsatellite alterations at 12 different loci, but found no evidence for microsatellite instability. However, AML is a heterogeneous group of disorders with respect to both clinical features and mechanisms of leukaemogenesis, so we cannot exclude the possibility that microsatellite instability may be found in rare cases. LOH is frequently observed in solid tumours and may indicate sites of tumour-suppressor genes involved in tumorigenesis. We observed LOH in only 3/20 AML patients (Table I), suggesting that the loci investigated play no consistent role in leukaemogenesis of AML.

**Acknowledgements**
This work was supported by the Leukaemia Research Fund and the Kay Kendall Leukaemia Trust. HS received a grant from the Austrian 'Fonds zur Förderung der wissenschaftlichen Forschung'.

### References

AALTONEN LA, PELTOMAKI P, LEACH FS, SISTONEN P, PYLKKANEN L, MECKLIN JP, JARVINEN H, POWELL SM, JEN J, HAMILTON SR, PETERSEN GM, KINZLER KW, VOGELSTEIN B AND DE LA CHAPELLE A. (1993). Clues to the pathogenesis of familial colorectal cancer. *Science*, **260**, 812–816.

BRONNER CE, BAKER SM, MORRISON PT, WARREN G, SMITH LG, LESCOE MK, KANE M, EARABINO C, LIPFORD J, LINDBLOM A, TANNERGARD P, BOLLAG RJ, GODWIN AR, WARD DC, NORDENSJOLD M, FISHEL R, KOLODNER R AND LISKAY M. (1994). Mutation in the DNA mismatch repair gene homologue hMLH1 is associated with hereditary non-polyposis colon cancer. *Nature*, **368**, 258–261.

FISHEL R, LESCOE MK, RAO MR, COPELAND NG, JENKINS NA, GARBER J, KANE M AND KOLODNER R. (1994). The human mutator gene homolog MSH2 and its association with hereditary nonpolyposis colon cancer. *Cell*, **77**, 167.

FONG KM, ZIMMERMANN PV AND SMITH PJ. (1995). Microsatellite instability and other molecular abnormalities in non-small cell lung cancer. *Cancer Res.*, **55**, 28–30.

FUJII H AND SHIMADA T. (1989). Isolation and characterization of cDNA clones derived from the divergently transcribed gene in the region upstream from the human dihydrofolate reductase gene. *J. Biol. Chem.*, **264**, 10057–10064.

GAO X, ZACHAREK A, SALKOWSKI A, GRIGNON DJ, SAKR W, PORTER AT AND HONN KV. (1995). Loss of heterozygosity of the BRCA1 and other loci on chromosome 17q in human prostate cancer. *Cancer Res.*, **55**, 1002–1005.

GONZALEZ-ZULUETA M, RUPPERT MJ, TOKINO K, TSAI YC, SPRUCK III CH, MIYAO N, NICHOLS PW, HERMANN GG, HORN T, STEVEN K, SUMMERHAYES IC, SIDRANSKY D AND JONES PA. (1993). Microsatellite instability in bladder cancer. *Cancer Res.*, **53**, 5620–5623.

HERNANDEZ JA, LAND KJ AND MCKENNA RW. (1995). Leukemias, myeloma, and other lymphoreticular neoplasms. *Cancer*, **75**, 381–394.

JONES MH, YAMAKAWA K AND NAKAMURA Y. (1992). Isolation and characterization of 19 dinucleotide repeat polymorphisms on chromosome 3p. *Hum. Mol. Genet.*, **1**, 131–133.

LEACH FS, NICOLAIDES NC, PAPADOPOULOS N, LIU B, JEN J, PARSONS R, PELTOMAKI P, SISTONEN P, AALTONEN LA, NYSTROM LAHTI M, GUAN XY, ZHANG J, MELTZER PS, YU JW, KAO FT, CHEN DJ, CEROSALETTI KM, FOURNIER REK, TODD S, KEWIS T, LEACH RJ, NAYLOR SL, WEISSENBACH J, MECKLIN JP, JARVINEN H, PETERSEN GM, HAMILTON SR, GREEN J, JASS J, WATSON P, LYNCH HT, TRENT JM, DE LA CHAPELLE A, KINZLER KW AND VOGELSTEIN B. (1993). Mutations of a mutS homolog in hereditary nonpolyposis colorectal cancer. *Cell*, **75**, 1215–1225.

LIU B, PARSONS RE, HAMILTON SR, PETERSEN GM, LYNCH HT, WATSON P, MARKOWITZ S, WILLSON JK, GREEN J, DE LA CHAPELLE A, KINZLER KW AND VOGELSTEIN B. (1994). hMSH2 mutations in hereditary nonpolyposis colorectal cancer kindreds. *Cancer Res.*, **54**, 4590–4594.

PAPADOPOULOS N, NICOLAIDES NC, WEI YF, RUBEN SM, CARTER KC, ROSEN CA, HASELTINE WA, FLEISCHMANN RD, FRASER CM, ADAMS MD, VENTER JC, HAMILTON SR, PETERSEN GM, WATSON P, LYNCH HT, PELTOMAKI P, MECKLIN JP, DE LA CHAPELLE A, KINZLER KW AND VOGELSTEIN B. (1994). Mutation of a mutL homolog in hereditary colon cancer. *Science*, **263**, 1625–1629.

PEIFFER SL, HERZOG TJ, TRIBUNE DJ, MUTCH DG, GERSELL DJ AND GOODFELLOW PJ. (1995). Allelic loss of sequences from the long arm of chromosome 10 and replication errors in endometrial cancers. *Cancer Res.*, **55**, 1922–1926.

RABBITTS TH. (1994). Chromosomal translocations in human cancers. *Nature*, **372**, 143–149.

ROBLEDO M, MARTINEZ B, ARRANZ E, TRUJILLO MK, GONZALEZ AGEITOS A, RIVAS C AND BENITEZ J. (1995). Genetic instability of microsatellites in hematological neoplasms. *Leukemia*, **9**, 960–964.

SILLY H, CHASE A, MILLS KI, APFELBECK U, SORMANN S, GOLDMAN JM AND CROSS NCP. (1994). No evidence for microsatellite instability or consistent loss of heterozygosity at selected loci in chronic myeloid leukaemia blast crisis. *Leukemia*, **8**, 1923–1928.

SPIRIO L, NELSON L, JOSLYN G, LEPPERT M AND WHITE R. (1992). A CA repeat 30–70 Kb downstream from the adenomatous polyposis coli (APC) gene. *Nucleic Acids Res.*, **20**, 642.

TAMURA G, SAKATA K, MAESAWA C, SUZUKI Y, TERASHIMA M, SATOH K, SEKIYAMA S, SUZUKI A, EDA Y AND SATODATE R. (1995). Microsatellite alterations in adenoma and differentiated adenocarcinoma of the stomach. *Cancer Res.*, **55**, 1933–1936.

THIBODEAU SN, BREN G AND SCHAID D. (1993). Microsatellite instability in cancer of the proximal colon. *Science*, **260**, 816–819.

WADA C, SHIONOYA S, FUJINO Y, TOKUHIRO H, AKAHOSHI T, UCHIDA T AND OHTANI H. (1994). Genomic instability of microsatellite repeats and its association with the evolution of chronic myelogenoous leukemia. *Blood*, **83**, 3449–3456.

WOOSTER R, CLETON-JANSEN A-M, COLLINS N, MANGION J, CORNELIS RS, COOPER CS, GUSTERSON BA, PONDER BAJ, VON DEIMLING A, WIESTLER OD, CORNELISSE CJ, DEVILEE P AND STRATTON MR. (1994). Instability of short tandem repeats (microsatellites) in human cancers. *Nature Genet.*, **6**, 152–156.